# Characterization and modulation of endoplasmic reticulum stress response target genes in *Kluyveromyces marxianus* to improve secretory expressions of heterologous proteins

**DOI:** 10.1186/s13068-021-02086-7

**Published:** 2021-12-14

**Authors:** Tianfang Shi, Jungang Zhou, Aijuan Xue, Hong Lu, Yungang He, Yao Yu

**Affiliations:** 1 State Key Laboratory of Genetic Engineering, School of Life Sciences, Fudan University, Shanghai, 200438 China; 2 Shanghai Engineering Research Center of Industrial Microorganisms, Shanghai, 200438 China; 3 Institutes of Biomedical Sciences, Fudan University, Shanghai, 200438 China

**Keywords:** *Kluyveromyces marxianus*; endoplasmic reticulum stress, Unfolded protein response, Overexpression, Lignocellulolytic enzyme

## Abstract

**Background:**

*Kluyveromyces marxianus* is a promising cell factory for producing bioethanol and that raised a demand for a high yield of heterologous proteins in this species. Expressions of heterologous proteins usually lead to the accumulation of misfolded or unfolded proteins in the lumen of the endoplasmic reticulum (ER) and then cause ER stress. To cope with this problem, a group of ER stress response target genes (ESRTs) are induced, mainly through a signaling network called unfolded protein response (UPR). Characterization and modulation of ESRTs direct the optimization of heterologous expressions. However, ESRTs in *K. marxianus* have not been identified so far.

**Results:**

In this study, we characterized the ER stress response in *K. marxianus* for the first time, by using two ER stress-inducing reagents, dithiothreitol (DTT) and tunicamycin (TM). Results showed that the Kar2–Ire1–Hac1 pathway of UPR is well conserved in *K. marxianus*. About 15% and 6% of genes were upregulated during treatment of DTT and TM, respectively. A total of 115 upregulated genes were characterized as ESRTs, among which 97 genes were identified as UPR target genes and 37 UPR target genes contained UPR elements in their promoters. Genes related to carbohydrate metabolic process and actin filament organization were identified as new types of UPR target genes. A total of 102 ESRTs were overexpressed separately in plasmids and their effects on productions of two different lignocellulolytic enzymes were systematically evaluated. Overexpressing genes involved in carbohydrate metabolism, including *PDC1*, *PGK* and *VID28*, overexpressing a chaperone gene *CAJ1* or overexpressing a reductase gene *MET13* substantially improved secretion expressions of heterologous proteins. Meanwhile, overexpressing a novel gene, *KLMA_50479* (named *ESR1*), as well as overexpressing genes involved in ER-associated protein degradation (ERAD), including *HRD3*, *USA1* and*YET3*, reduced the secretory expressions. *ESR1* and the aforementioned ERAD genes were deleted from the genome. Resultant mutants, except the *yet3*Δ mutant, substantially improved secretions of three different heterologous proteins. During the fed-batch fermentation, extracellular activities of an endoxylanase and a glucanase in *hrd3*Δ cells improved by 43% and 28%, respectively, compared to those in wild-type cells.

**Conclusions:**

Our results unveil the transcriptional scope of the ER stress response in *K. marxianus* and suggest efficient ways to improve productions of heterologous proteins by manipulating expressions of ESRTs.

**Supplementary Information:**

The online version contains supplementary material available at 10.1186/s13068-021-02086-7.

## Background

Protein secretion is initiated from the rough endoplasmic reticulum (ER) lumen, where nascent polypeptides are bound by ER-resident proteins for correct folding and processing. Only properly folded and assembled proteins are exported from the ER to the Golgi for further modification, before being transported to the extracellular space, vacuoles or other organelles [1]. When the ER encounters a high flux of heterologous proteins, its folding capacity could be transiently saturated, thus leading to the accumulation of misfolded or unfolded proteins and causing ER stress [2]. ER stress causes substantial transcriptomic changes. For instance, 7.8% of genes in *Neurospora crassa* and 6.8% of genes in *Saccharomyces cerevisiae* were upregulated by both dithiothreitol (DTT) and tunicamycin (TM), while 6.8% of genes in *Komagataella phaffii* were upregulated by both DTT and the overexpression of *HAC1* [3–5]. ER stress response target genes (ESRTs) were defined as genes induced upon ER stress and were involved in the response to cope with the stress. A proportion of ESRTs was induced through a signaling network called unfolded protein response (UPR), which is one of the best-characterized pathways to deal with ER stress [6]. Meanwhile, some ESRTs were induced independently of UPR, as reported in *N. crassa* and *S. cerevisiae* [4, 7].

The UPR network is composed of stress sensors, transcriptional activators and downstream target genes. Three branches of UPR were identified and named after sensors, including IRE1 (inositol-requiring enzyme 1), PERK (protein kinase RNA-like endoplasmic reticulum kinase) and ATF6 (activating transcription factor 6) [6]. In yeast, UPR regulation solely depends on the most conserved IRE1 branch. In *S. cerevisiae*, Ire1 may be dissociated from the ER-luminal resident chaperone Kar2 (Bip) upon ER stress. The released Ire1 recognizes misfolded proteins, triggers Ire1 oligomerization and then activates themselves to splice the *HAC1* mRNA into the translationally competent *HAC1* mRNA [8]. Then, the spliced *HAC1* mRNA is translated into a transcriptional activator that recognizes the specific DNA sequences, called UPR elements (UPRE), to induce transcriptions of UPR target genes [9]. UPR target genes function from protein folding, phospholipid synthesis, protein translocation, glycosylation and vacuolar transport to ER-associated degradation (ERAD) [3].

Naturally, components of the UPR network can serve as targets for optimization to improve the secretory expressions of heterologous proteins. In *Aspergillus niger* var. *awamori*, overexpression of the activated UPR transcription factor HacA constitutively induced the UPR pathway and enhanced productions of both *Trametes versicolor* laccase and bovine preprochymosin [10]. ER-resident chaperones, including Kar2, Lhs1 and Jem1, and protein disulfide isomerase Pdi1, are classic UPR targets [3]. The introduction of an extra copy of *KAR2* into *S. cerevisiae* caused more than a 20-fold increase in the amount of extracellular prochymosin [11]. Similarly, overexpression of *KAR2* in *K. phaffii* increased the secretion of a single-chain antibody fragment [12]. In *S. cerevisiae*, overexpression of *LHS1* or *JEM1* significantly increased the secretory expression of recombinant human albumin [13]. Overexpressing *S. cerevisiae* disulfide isomerase Pdi1 in *K. phaffii* increased the secretory yield of human parathyroid hormone even though it does not contain any cysteine residue [14]. In addition, UPR-independent ESRTs can also be modified to improve secretion. *RES-1* encodes an *N. crassa* transcription factor that responds to intracellular calcium disturbances induced by the ER stress. *RES-1* was induced independently of Ire1 or Hac1 and deletion of *RES-1* elevated the amount of secreted cellulase by 50% [4].

*Kluyveromyces marxianus* is a homothallic hemiascomycetous yeast species commonly isolated in dairy products, grape, papaya and Mexican fermented corn dough [15]. It is the fastest-growing eukaryote known so far, and can assimilate inulin, lactose and pentose (e.g., xylose and arabinose) that cannot be utilized by the traditional ethanologenic yeast *S. cerevisiae*. It is noteworthy that *K. marxianus* exhibits weak glucose repression that is preferable for the fermentation of mixed sugars such as hemicellulose hydrolysate [16]. Beyond that, *K. marxianus* is highly thermotolerant, as it can grow at a temperature up to 52 °C. Given its desirable traits, *K*. *marxianus* is considered a promising host for the productions of heterologous proteins and bioethanol [17].

So far, ER stress response and UPR pathway have not been characterized in *K. marxianus*, which hinders the improvement of heterologous proteins expressions by modulating ESRTs. In this study, we showed that the Kar2–Ire1–Hac1 pathway of UPR was well conserved in *K. marxianus*. During treatments of ER stress-inducing reagents DTT and TM, 15.1% and 6.4% of genes were upregulated, respectively. A total of 115 upregulated genes were identified as ESRTs, among which 97 genes were characterized as UPR target genes. Effects of the overexpression or deletion of ESRT on productions of heterologous proteins were systematically evaluated. Overexpression of genes involved in carbohydrate metabolism, chaperone gene and reductase gene improved the secretory expression. Meanwhile, deletions of genes involved in ERAD improved the secretory expressions. Our results revealed the transcriptional scope of ER stress response in *K. marxianus* and identified valuable target genes to be engineered to improve expressions of heterologous proteins.

## Results

### Upstream components of UPR pathway in *K. marxianus*

**Fig. 1:**
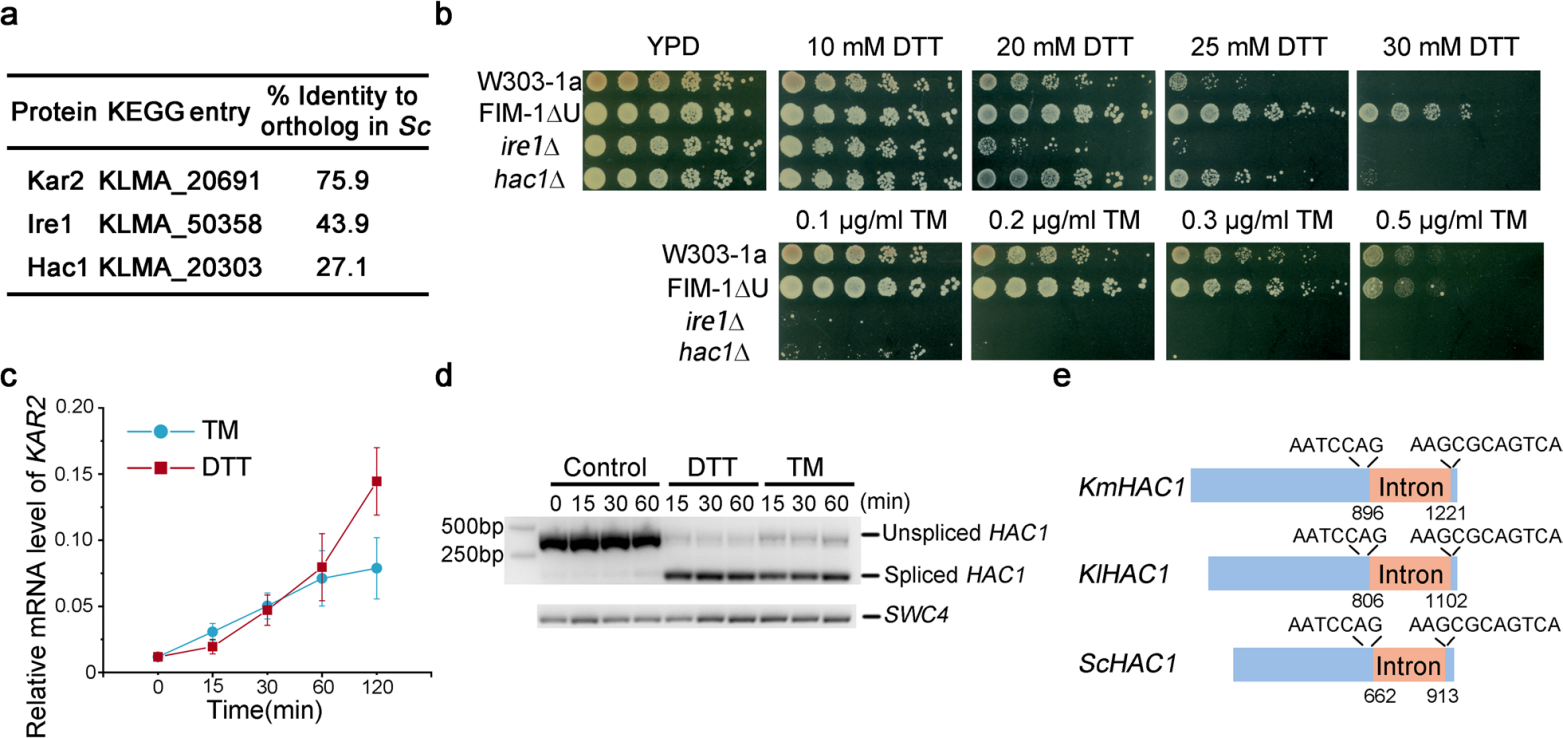
Identification of upstream components of the UPR pathway in *K. marxianus*. **a** Identities of Kar2, Ire1 and Hac1 in *K. marxianus* to their orthologs in *S. cerevisiae* (Sc). **b** Sensitivity of W303-1a, FIM-1ΔU, *ire1*Δ and *hac1*Δ mutants to ER stress-inducing reagents. Cells were diluted fivefold and dilutions were spotted onto YPD containing different concentrations of DTT or TM. **c** Induction of *KAR2* upon treatment of DTT and TM. Exponentially growing cells were treated with DTT and TM for indicated times. mRNA level of *KAR2* relative to 18s rDNA was measured. The value represented mean ± SD (*n* = 3). **d** Splicing of *HAC1* upon treatment of DTT and TM. Sizes of the product representing unspliced and spliced forms of *HAC1* were 443 bp and 117 bp, respectively. Cells were treated as in **c**. *SWC4* was detected as a loading control. **e** Schematic representation of *HAC1* introns in *K. marxianus*, *K. lactis* and *S. cerevisiae*

In the upstream processes of the UPR pathway, Kar2, Ire1 and Hac1 cooperate to sense unfolded or misfolded proteins, to trigger unfolded protein response and maintain the homeostasis in the endoplasmic reticulum [18]. Orthologs of these three proteins were identified in *K. marxianus* based on sequence similarity (Fig. 1a). Hac1 shares poor sequence identity with its ortholog in *S. cerevisiae*, while Kar2 is relatively well conserved.

DTT and TM are two classic ER stress-inducing reagents. DTT blocks disulfide-bond formation and TM inhibits N-linked glycosylation, both leading to the disruption of protein folding in the ER [19]. As shown in Fig. 1b, 25 mM DTT caused severe growth defect of a wild-type *S. cerevisiae* strain W303-1a, while wild-type *K. marxianus* strain FIM-1ΔU strain was able to grow in the presence of 30 mM DTT. The result suggests *K. marxianus* is more tolerant to DTT stress than *S. cerevisiae*. The sensitivity of *K. marxianus* and *S. cerevisiae* to TM were similar, as FIM-1ΔU and W303-1a cells exhibited growth defects in the presence of 0.5 μg/mL TM (Fig. 1b). To verify the roles of Ire1 and Hac1 in ER stress in *K. marxianus*, *IRE1* and *HAC1* were knocked out by CRISPR separately and growth of these two mutants was examined in the medium containing DTT or TM. As shown in Fig. 1b, the growth of *ire1*Δ and *hac1*Δ mutants was significantly impaired in the presence of 0.1 μg/mL TM. The *ire1*Δ mutant was more sensitive to DTT than the *hac1*Δ mutant, as *ire1*Δ and *hac1*Δ cells exhibited severe growth defects in the presence of 25 mM and 30 mM DTT, respectively. The result suggests that Ire1 and Hac1 maintain homeostasis of the ER to reduce deleterious effects caused by the ER stress in *K. marxianus*.

In *S. cerevisiae, KAR2* is an essential gene and transcription of *KAR2* is rapidly induced upon DTT or TM treatment, which is a hallmark event of UPR response [20, 21]. We failed to obtain the null mutant of *KAR2* in *K. marxianus*, suggesting that *KAR2* is also an essential gene in *K. marxianus*. Similar inductions of *KAR2* after being treated with ER stress-inducing reagents were observed in *K. marxianus*. In the presence of 10 mM DTT or 0.5 μg/mL TM, the increases of the *KAR2* mRNA levels were quite similar, which were about 50-fold after 30 min and 75-fold after 60 min. After 120 min, the level of *KAR2* induced by DTT increased by 150-fold, which was twice that induced by TM (Fig. 1c). The results suggest prolonged incubation with DTT induces an acuter ER stress than with TM.

In *S. cerevisiae*, ER stress triggers Ire1-mediated splicing of *HAC1* mRNA [8]. In *K. marxianus*, the ORF of *HAC1* is 912-bp in length. Few spliced *HAC1* mRNA was detected in cells in normal conditions (Fig. 1d). In contrast, splicings of *HAC1* mRNA were rapidly induced after adding DTT or TM, leading to the appearance of a small fragment in the RT-PCR assay (Fig. 1d). Sequence analyses of both the unspliced and spliced forms of *HAC1* revealed that a 325-bp intron was removed. As shown in Fig. 1e, cleavage motifs before and after exon–intron junctions were the same among *K. marxianus*, *K. lactis* and *S. cerevisiae*, suggesting that a conserved *HAC1*-splicing mechanism exists within Saccharomycetaceae.

### Transcriptomic analysis of *K. marxianus* during ER stress

**Fig. 2:**
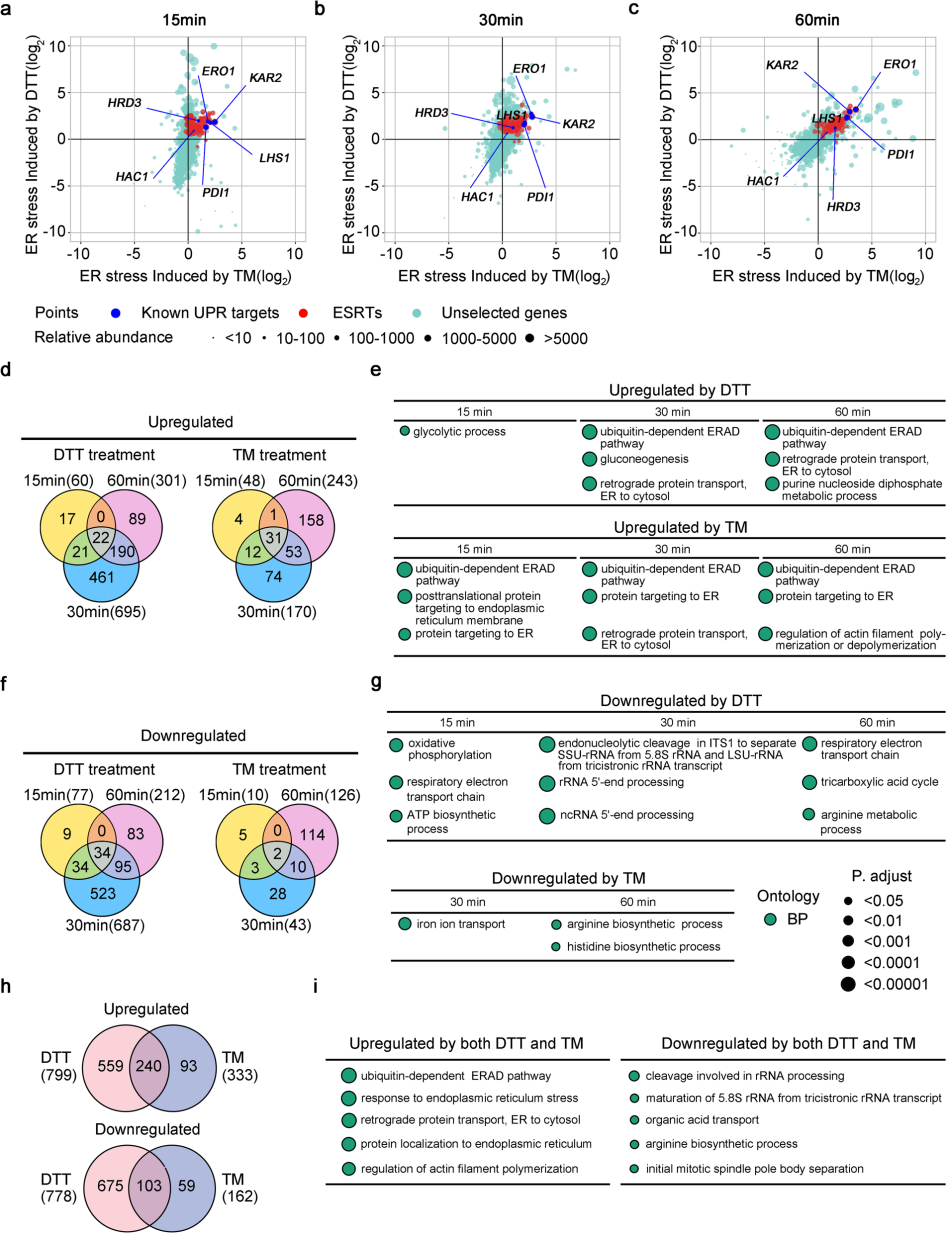
Identification of differentially expressed genes during ER stress. Comparison of ER stress induced by DTT and TM. Exponentially growing cells were treated with DTT or TM for 15 (**a**), 30 (**b**) and 60 min (**c**). Induction by ER stress was calculated by dividing the average abundance of a gene in cells treated with DTT or TM by that in cells without treatment. At the indicated time point, fold change (Log_2_) of a certain gene upon DTT treatment was plotted against that upon TM treatment in the chart. Points representing ESRTs were red. Points corresponding to several known UPR target genes were blue. Points representing other genes were green. The size of the point reflected the mean of FPKM value of a certain gene after treatment of DTT and TM. **d**, **f**, **h** Venn diagrams of upregulated and downregulated genes after TM and DTT treatment. Genes significantly upregulated (Fc > 2, *q* value < 0.1) or downregulated (Fc < 0.5, *q* value < 0.1) upon drug treatment were counted. The number of upregulated or downregulated genes in indicated conditions is shown in the parenthesis. **e**, **g**, **i** GO enrichment analysis of differentially expressed genes during ER stress. The enrichment analysis was based on the biological process (BP). Top-ranked terms containing 10 ~ 50 genes and with an adjusted *P*-value < 0.05 are listed in **e**, **g**. Top-ranked terms containing 10 ~ 100 genes and with an adjusted *P*-value < 0.05 are listed in **i**. The size of a point represented the adjusted *P*-value. A full list of enriched GO terms is shown in Additional file 3: Table S3

To analyze the transcriptional scope of ER stress in *K. marxianus*, wild-type FIM-1ΔU cells were grown in the presence of 10 mM DTT or 0.5 µg/mL TM for 15, 30 and 60 min before they were collected and subjected to RNAseq (Additional file 1: Table S1). The expression level of a gene in the treated sample at each time point was compared with that in the untreated control. As shown in Fig. 2a–c, several classic UPR target genes, including *HAC1*, *KAR2*, *PDI1*, *ERO1* and *HRD3*, were substantially upregulated during the treatment of DTT or TM. The result suggests that ER stress and UPR in *K. marxianus* are invoked after adding DTT or TM.

FIM-1ΔU strain harbors 5202 genes in total [22]. After a 15-min treatment of DTT, 60 genes were significantly upregulated (Fold change > 2, q value < 0.1), among which genes involved in the glycolytic process were enriched (Fig. 2d, e, Additional file 2: Table S2), while 77 genes were significantly downregulated, among which genes involved in the oxidative phosphorylation were enriched (Fig. 2f, g, Additional file 2: Table S2). The results suggest a shift from respiration to glycolysis during the initial response to DTT. After a 30-min treatment of DTT, 695 genes were significantly upregulated, among which genes involved in ERAD and retrograde protein transport were enriched (Fig. 2d, e). Meanwhile, 687 genes were significantly downregulated, among which genes involved in rRNA processing were enriched, suggesting the repression of translation (Fig. 2f, g). Reduction of the rRNA processing probably is not a specific response to DTT, as a transient reduction in transcripts for the translation apparatus was observed in *S. cerevisiae* during various environmental stresses, including heat shock, acid, alkali, H_2_O_2_, salt and sorbitol [23]. After a 60-min treatment of DTT, 301 and 212 genes were upregulated and downregulated, respectively (Fig. 2d, f). At this time point, cells kept the upregulation of genes involved in ERAD and retrograde protein transport, while genes involved in respiration, TCA cycles and arginine metabolic process were downregulated (Fig. 2e, g).

During the treatment of TM, 48, 170 and 243 genes were significantly upregulated after 15, 30 and 60 min, respectively (Fig. 2d, Additional file 2: Table S2). Among upregulated genes, genes involved in the processes of ERAD and protein targeting to ER were enriched (Fig. 2e). Interestingly, genes involved in the regulation of actin filament polymerization were upregulated after 60 min (Fig. 2e), composing a new type of genes induced upon ER stress. During the treatment of TM, 10, 43 and 126 genes were significantly downregulated after 15, 30 and 60 min, respectively (Fig. 2f). Among the downregulated genes, genes involved in iron ion transport, arginine and histidine biosynthesis were enriched (Fig. 2g, Additional file 2: Table S2). Downregulated iron ion transport might reduce the metabolic and respiratory activity of mitochondria [24], leading to the repression of respiration as shown in cells treated with DTT. Downregulation of amino acid biosynthesis might be related to the repression of the translation of certain target proteins [25].

In general, 15.2% of genes and 14.9% of genes were upregulated and downregulated, respectively, after DTT treatment, while 6.4% and 3.1% of genes were upregulated and downregulated after TM treatment. As shown in Additional file 11: Fig. S1, 15-min and 30-min DTT treatment caused a larger magnitude of changes in the expressed transcripts than 15-min, 30-min and 60-min TM treatment. These results indicated that in the current concentrations of both chemicals, DTT induced an acuter ER stress than TM. In total, 16.9% and 15.9% of genes were upregulated and downregulated, respectively, after treatment of at least one ER stress-inducing reagents, suggesting that ER stress leads to a profound transcriptomic change in *K. marxianus*. A total of 240 genes (4.6% of total genes) were upregulated by both DTT and TM. Since DTT and TM caused ER stress through different mechanisms, these genes were likely to be induced by misfolding of proteins in ER rather than other effects (Fig. 2h and Additional file 2: Table S2). Among the co-induced genes, genes involved in ERAD, ER protein transport and localization, and actin filament organization were enriched (Fig. 2i). A total of 103 genes were downregulated by both DTT and TM, among which genes involved in rRNA processing, organic acid transport and arginine biosynthesis were enriched (Fig. 2i).

### Identification of UPR target genes and UPRE in *K. marxianus*

To determine ESRT and UPR target genes, homologues of 12 classic UPR target genes were selected as reference genes [19], including genes encoding chaperone proteins (*KAR2, LHS1*), genes involved in the disulfide bond formation (*PDI1*, *ERO1*, *MPD1*), genes involved in the vesicle transport (*SFB3*, *SEC24*, *SEC12*) and genes involved in ERAD (*DER1*, *HRD1*, *HRD3*, *UBC7*). All reference genes were induced both by DTT and TM. Analyses of the transcriptional profiles of the 12 reference genes revealed four different patterns of induction during the treatment of DTT and TM. Each pattern was represented by a group of reference genes. A given gene displaying a similar transcriptional pattern to those of representative reference genes of a certain group was identified as an ESRT and classified into the corresponding group. By analyzing the transcriptomic data of *hac1*Δ cells, ESRTs whose inductions were dependent on Hac1 were identified as UPR target genes (see detail in “Methods” section).

**Fig. 3:**
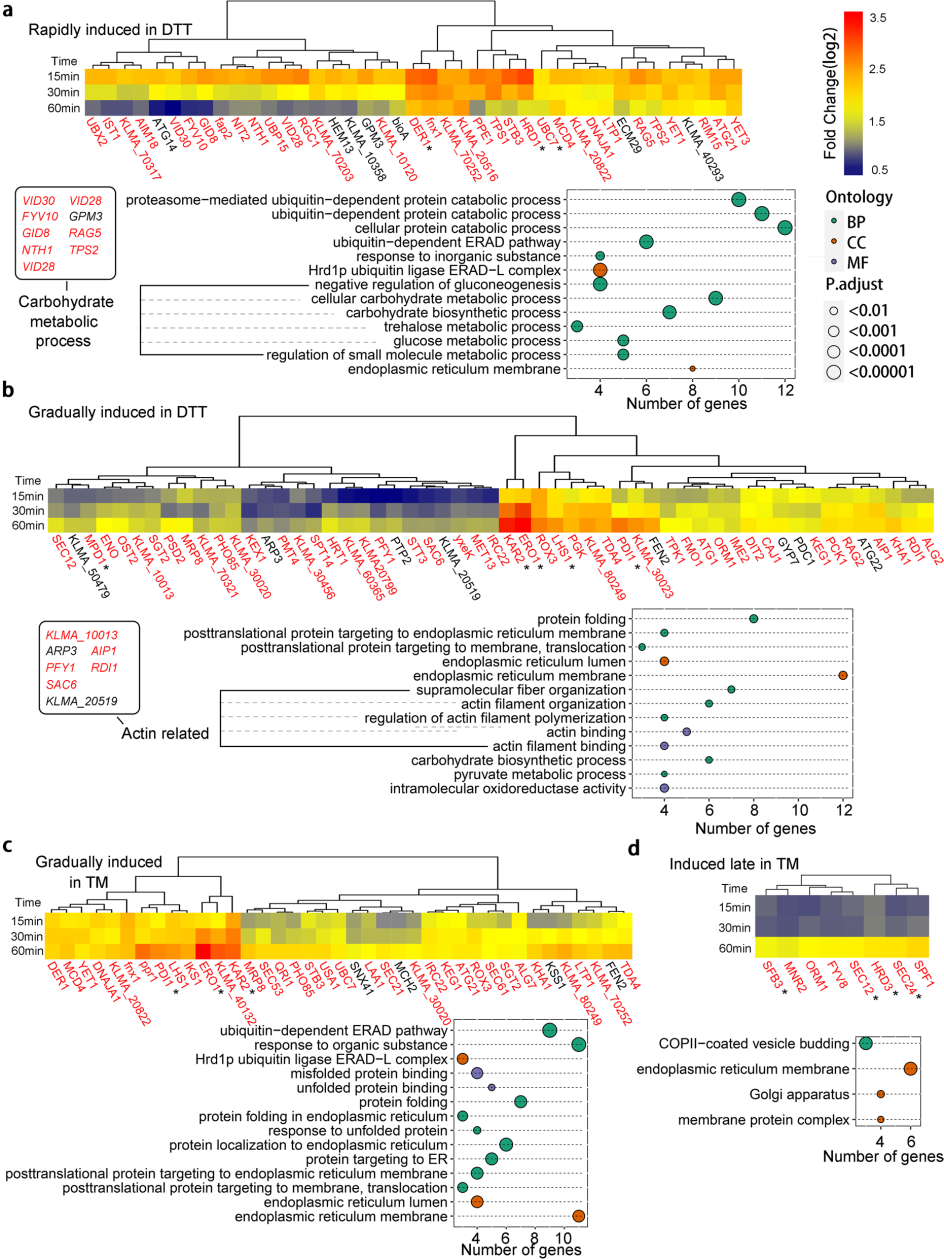
ESRTs clustered in 4 groups. **a**-**d** Significantly upregulated genes during DTT or TM treatment were clustered in 4 groups. Characteristics of transcriptional patterns in each group were defined by reference genes (marked by asterisks). Genes displaying similar transcriptional patterns to those of reference genes were clustered together. Genes rapidly induced in DTT are shown in **a**. Genes gradually induced in DTT are shown in **b**. Genes gradually induced in TM are shown in **c**. Genes induced late in TM are shown in **d**. UPR target genes are labeled in red. GO enrichment of genes in each group are shown below the cluster. The colour of a point represented the ontology. BP stood for biological process, CC for cellular component and MF for molecular function. The size of a point represented the adjusted *P*-value

Genes in the first group were rapidly induced upon DTT treatment. Reference genes were *DER1*, *HRD1* and *UBC7*. Der1 and Hrd1 are the subunits of the Hrd1 complex, an essential component of ERAD, while Ubc7 is an E2 interacting with the Hrd1 complex [26]. Expression levels of genes in this group reached the peak after 15 min upon DTT treatment and then gradually went down at the following time points. A total of 41 ESRTs, including 34 UPR target genes, were included in this group. Since all three reference genes were related to ERAD, it was expected to obtain the enrichment of terms associated with ubiquitin-dependent protein catabolic process. 9 genes involved in the carbohydrate metabolic process were enriched in this group, which included 4 genes (*VID28*, *VID30*, *FYV10* and *GID8*) encoding subunits of the glucose-induced degradation deficient (GID) complex (Fig. 3a). The GID complex is responsible for the repression of gluconeogenesis [27]. Among the 9 genes, *GPM3* was not designated as a UPR target gene and *RAG5* (homolog of *ScHXK2*) was previously identified as a UPR target gene in *S. cerevisiae* [3], while the rest 7 genes were linked with UPR for the first time. *UBC7*, a reference gene of this group, did not pass the statistical criterion of UPR target genes (P = 0.71). The upregulation of *UBC7* by 15-min DTT treatment was slightly but not significantly reduced in *hac1*Δ cells (Additional file 1: Table S1). A similar situation was reported in *S*. *cerevisiae*, as *UBC7* was just below the criterion of the UPR target gene in a transcriptomic analysis [19]. However, *UBC7* was still identified as a UPR target gene in the same report, probably due to its role in ERAD [19]. In another report, Hac1-dependent upregulation of *UBC7* was proved experimentally in *S*. *cerevisiae* [28], suggesting *UBC7* was a false negative in the transcriptomic analysis. Therefore, *UBC7* was designated as a UPR target gene of *K*. *marxianus*.

Genes of the second group were induced gradually upon treatment of DTT. *KAR2*, *LHS1*, *ERO1*, *MPD1* and *PDI1* were reference genes of this group. A total of 55 genes fit the criterion, including 47 UPR target genes. GO analysis revealed that 7 genes involved in the actin filament organization were enriched in this group (Fig. 3b). None of these 7 genes was directly linked with ER stress or UPR before. However, genes related to actin filament organization, including *RDI1*, were overexpressed in an ethanol-tolerant *S*. *cerevisiae* strain during ethanol stress, while UPR target genes, including *HAC1*, *ERO1* and *KAR2* were also upregulated [29]. Secretory vesicles are transported on actin filaments [30]. Therefore, the induction of genes involved in actin filament organization might contribute to the relief of ER stress by promoting secretory vesicles transport.

During the treatment of TM, 39 genes, including reference genes *KAR2*, *ERO1* and *PDI1*, were gradually induced. A total of 35 UPR target genes were included in this group. Among 39 ESRTs, genes encoding ER proteins and genes involved in ERAD were enriched. In *S. cerevisiae* and *A. niger*, ER stress triggered the removal of unfolded proteins by the ERAD system [3, 31]. Our results suggest a conserved relationship between ER stress response and the ERAD in *K. marxianus*.

The fourth group represented genes induced late upon TM treatment. The levels of four reference genes, including *SEC12*, *SEC24*, *SFB3* and *HRD3* remained constant at 15 and 30 min and then were upregulated at 60 min. Another 4 ESRTs, including *FYV8, MNR2*, *SPF1* and *ORM1*, were included in this group. All 8 genes were identified as UPR target genes. *FYV8* was a gene with an unknown function. *MNR2* encodes a vacuolar membrane protein required for magnesium homeostasis [32]. *SPF1* encodes an ion transporter of the ER membrane [33]. *ORM1* encodes an ER membrane protein that mediates sphingolipid homeostasis [34]. Three reference genes, including *SEC12* [35], *SEC24* [36] and *SFB3* [37], encode proteins involved in the COP II-coated vesicle formation from the ER membrane. The other reference gene, *HRD3* encodes a subunit of the Hrd1 complex located in the ER membrane [38]. Since *MNR2*, *SPF1* and four reference genes were related to ER membrane, it was not surprising that the GO term of ER membrane was enriched in this group (Fig. 3d).

**Fig. 4:**
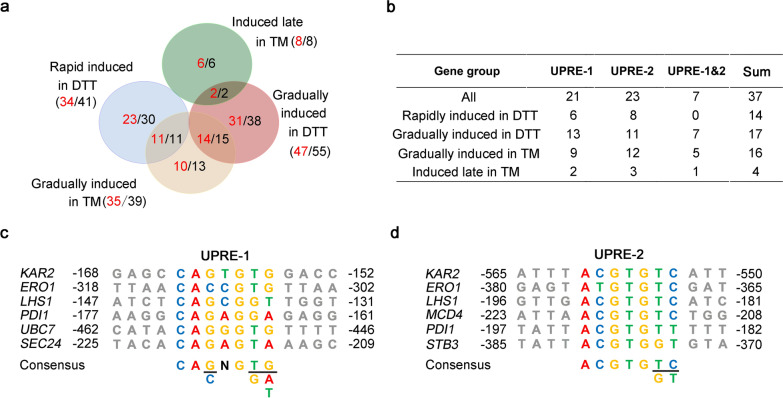
Identification of UPREs in the promoters of UPR target genes. **a** The Venn diagram of the overlapping of ESRTs and UPR target genes. The numbers of ESRTs and UPR target genes in each subset were labeled in black and red, respectively. **b** Distribution of UPRE-1 and UPRE-2 motifs in the UPR targets genes from different groups. **c**, **d** Alignment of promoters of UPR target genes that contain UPRE-1 (**c**) or UPRE-2 motifs (**d**). The consensus is listed below the alignment. Flanking nucleotides are indicated in light grey. Coordinates are relative to the start codon of the respective gene

There was an overlapping of ESRTs and UPR targets genes from the four groups discussed above (Fig. 4a, b). In total, 115 ESRTs and 97 UPR target genes were included in the four groups (Additional file 4: Table S4). Among 115 ESRTs, 67 genes were related to UPR or ER stress previously. The remaining 48 genes were linked with ER stress for the first time (Additional file 4: Table S4). Among 97 UPR targets, 62 genes were linked with UPR for the first time (Additional file 4: Table S4). Novel ESRTs and UPR target genes might underlie species-specific characteristics of the ER stress response and UPR network in *K. marxianus*.

In *S. cerevisiae*, Hac1 binds to UPRE motifs to initiate the expression of UPR target genes [39]. The promoters of UPR target genes in *K. marxianus* were analyzed to identify potential Hac1-binding sites. A 7-bp consensus (5ʹ-*CASNGKD*-3ʹ) resembled the core UPRE-1 motif (5ʹ-*CAGNGTG*-3ʹ) in *S. cerevisiae* (Fig. 4c). Another 7-bp consensus (5ʹ-*ACGTGKY*-3ʹ) exhibited high similarity to the UPRE-2 motif (5ʹ-*TACGTG*-3ʹ) in *S. cerevisiae* (Fig. 4d). In terms of sequence similarity, the UPRE-2 motif was more conserved than the UPRE-1 motif. Among 97 UPR target genes identified in this study, 14 genes contain a single UPRE-1 motif and 16 genes contain a single UPRE-2 motif in their promoters, suggesting these genes are induced by direct binding of Hac1 (Fig. 4b and Additional file 4: Table S4). Meanwhile, 7 genes contain both UPRE motifs (Fig. 4b and Additional file 4: Table S4). Notably, among homologues of the 7 genes in *S. cerevisiae*, *ERO1*, *LHS1* and *KAR2* contained UPRE-1 and UPRE-2 motifs. The result suggests that the dual-site recognition of *ERO1*, *LHS1* and *KAR2* by Hac1 may be conserved in *S. cerevisiae* and *K. marxianus* [39].

### Effects of overexpressing ESRTs on secretory expressions of lignocellulolytic enzymes

To study the role of ESRTs in regulating secretory expression, two strains constitutively secreting heterologous lignocellulolytic enzymes were constructed. RuCelA is a bifunctional xylanase/endoglucanase from yak rumen microorganisms that can simultaneously produce xylo-oligosaccharides and cello-oligosaccharides from lignocellulose [40]. AnFaeA is a feruloyl esterase from *A. niger*, which is a part of the hemicellulase complex that acts collectively and synergistically to completely hydrolyze feruloyl-polysaccharide. Genes encoding RuCelA and AnFaeA were integrated into the *INU1* loci of a T1 strain to obtain LHP1021 and LHP643, respectively. The T1 strain was derived from FIM-1ΔU that improved the yield of heterologous proteins by attenuating autophagy [41]. In LHP1021 and LHP643, RuCelA and AnFaeA were expressed by a strong *INU1* promoter and their secretions were directed by an alpha factor signal peptide from *S. cerevisiae*. RuCelA is a 532 aa protein containing 5 cysteine residues, while AnFaeA is a 282 aa protein containing 7 cysteine residues. Overexpression of these two heterologous enzymes might cause different types of ER stress, which provided a good system to evaluate the versatility of ESRTs in handling ER stress.

**Fig. 5:**
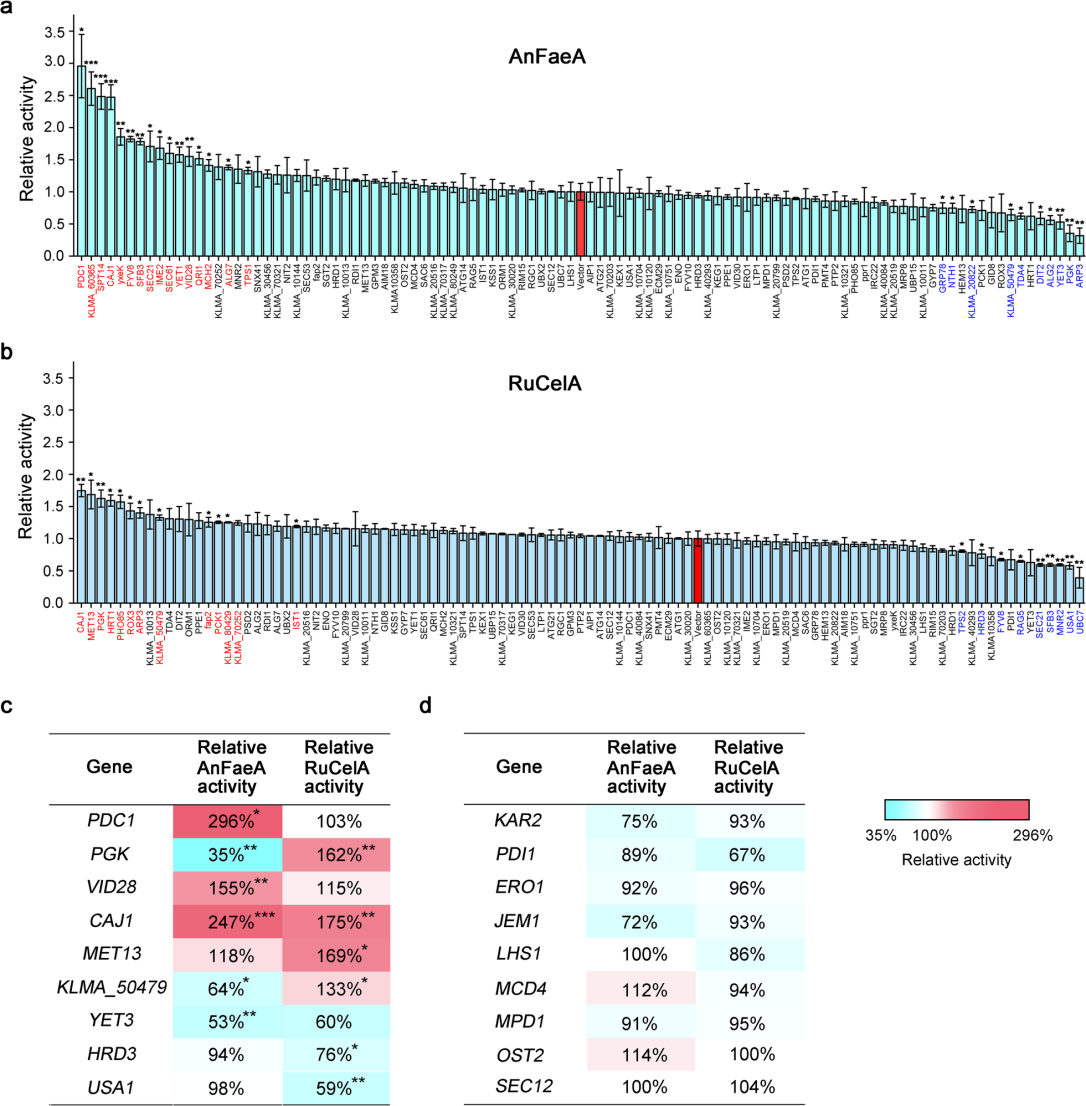
Effects of overexpressing ESRTs on secretory expressions of lignocellulolytic enzymes. Effects of overexpressing ESRTs on secretory expressions of AnFaeA (**a**) and RuCelA (**b**). AnFaeA or RuCelA was integrated at *INU1* loci in the genome. Overexpression of the ESRT was driven by a *TEF* promoter on a multi-copy plasmid. The activity of AnFaeA or RuCelA in the supernatant of cells transformed with a void vector was designated as unit 1. The column corresponding to the void vector control is in red. Value represented mean ± SD (*n* = 3) (*P < 0.05, **P < 0.01, ***P < 0.001). ESRTs that significantly improved and reduced extracellular activities are in red and blue, respectively. **c** Representative ESRTs that displayed significant effects on secretory expressions of lignocellulolytic enzymes (*P < 0.05, **P < 0.01, *** *P* < 0.001). **d** Effects of known UPR target genes on secretory expressions of lignocellulolytic enzymes

The ESRTs were inserted behind a strong *TEF* promoter separately on a multi-copy plasmid. The resultant plasmids were separately transformed into LHP1021 and LHP643. Except for 13 ESRTs that might cause cytotoxicity to *K. marxianus,* thus failing to obtain transformants, a total of 102 ESRTs were successfully overexpressed and effects on the secretory expressions of both AnFaeA and RuCelA were measured. As shown in Fig. 5a and b, overexpressing 16 and 11 ESRTs significantly increased the extracellular activities of AnFaeA and RuCelA, respectively. Overexpressing 10 and 9 ESRTs significantly reduced the secretory expressions of AnFaeA and RuCelA, respectively. Representative ESRTs that affected secretory expressions are listed in Fig. 5c. Due to the limited fundamental research in *K. marxianus*, functions of these ESRTs were mainly predicted by their orthologs in *S. cerevisiae*. *PDC1*, *PGK* and *VID28* are involved in carbohydrate metabolism. *PDC1* encodes a pyruvate decarboxylase isozymes that decarboxylates pyruvate to acetaldehyde [42]. Overexpression of *PDC1* caused the highest improvement for the secretory expression of AnFaeA. *PGK* encodes phosphoglycerate kinase [43]. Vid28 is a subunit of the GID complex, which is a highly conserved ubiquitin ligase complex targeting key enzymes of gluconeogenesis for degradation [27]. Overexpressing *PGK* and *VID28* significantly improved the expression of RuCelA and AnFaeA. Met13 is involved in the tetrahydrofolate interconversion pathway [44]. Overexpressing *MET13* significantly improved the expression of RuCelA. Caj1 is a chaperone that regulates the stability or turnover of plasma membrane proteins [45]. Out of the 102 ESRTs overexpressed in this study, *CAJ1* was the only gene that improved the secretory expressions of both AnFaeA and RuCelA. The result suggests that only a few ESRTs, when overexpressed, displayed positive effects on the productions of heterologous proteins in *K. marxianus*.

On the other hand, overexpressing several genes involved in ERAD significantly reduced the secretory expressions of AnFaeA or RuCelA. Yet3 is a homolog of human Bap31p which plays a role in targeting the misfolded protein to ERAD [46]. Secretory expressions of RuCelA and AnFaeA were reduced upon overexpressing *YET3*. Hrd3 and Usa1 belong to the Hrd1 complex which is responsible for the ubiquitination of ERAD-L substrates [47]. Overexpressing *HRD3* and *USA1* significantly reduced the expression of RuCelA.

In addition, overexpression of an unknown gene, *KLMA_50479* increased the extracellular expression of RuCelA but reduced the expression of AnFaeA. Since *KLMA_50479* was induced by ER stress and might play a role in regulating secretion, *KLMA_50479* was named *ESR1* (ER stress response gene 1).

In previous reports, overexpressing *KAR2*, *PDI1*, *ERO1, JEM1* or *LHS1*, promoted the productions of heterologous proteins [10, 12–14]. However, overexpressing these genes, as well as four other conserved UPR target genes, *MCD4*, *MPD1*, *OST2* and *SEC12,* exhibited no significant or very mild effects on the expressions of AnFaeA and RuCelA (Fig. 5d). The failure of improving secretion by overexpressing these genes in *K. marxianus* might be because choices of promoter and host in this study were different from those in previous studies, including *AOX1* promoter in *K. phaffii* [12, 14], *glaA* promoter in *A. niger* [10], *ADH1* and *PGK1* promoters in *S. cerevisiae* [13]. In addition, the properties of an esterase (AnFaeA) and a glycosidase (RuCelA) aimed for secretion in this study were different from those of proteins used previously, including protease, oxidoreductase [10], antibody fragment [12], human albumin and hormone [13, 14].

### Deletions of *ESR1* and ERAD genes improved secretory expressions

**Fig. 6:**
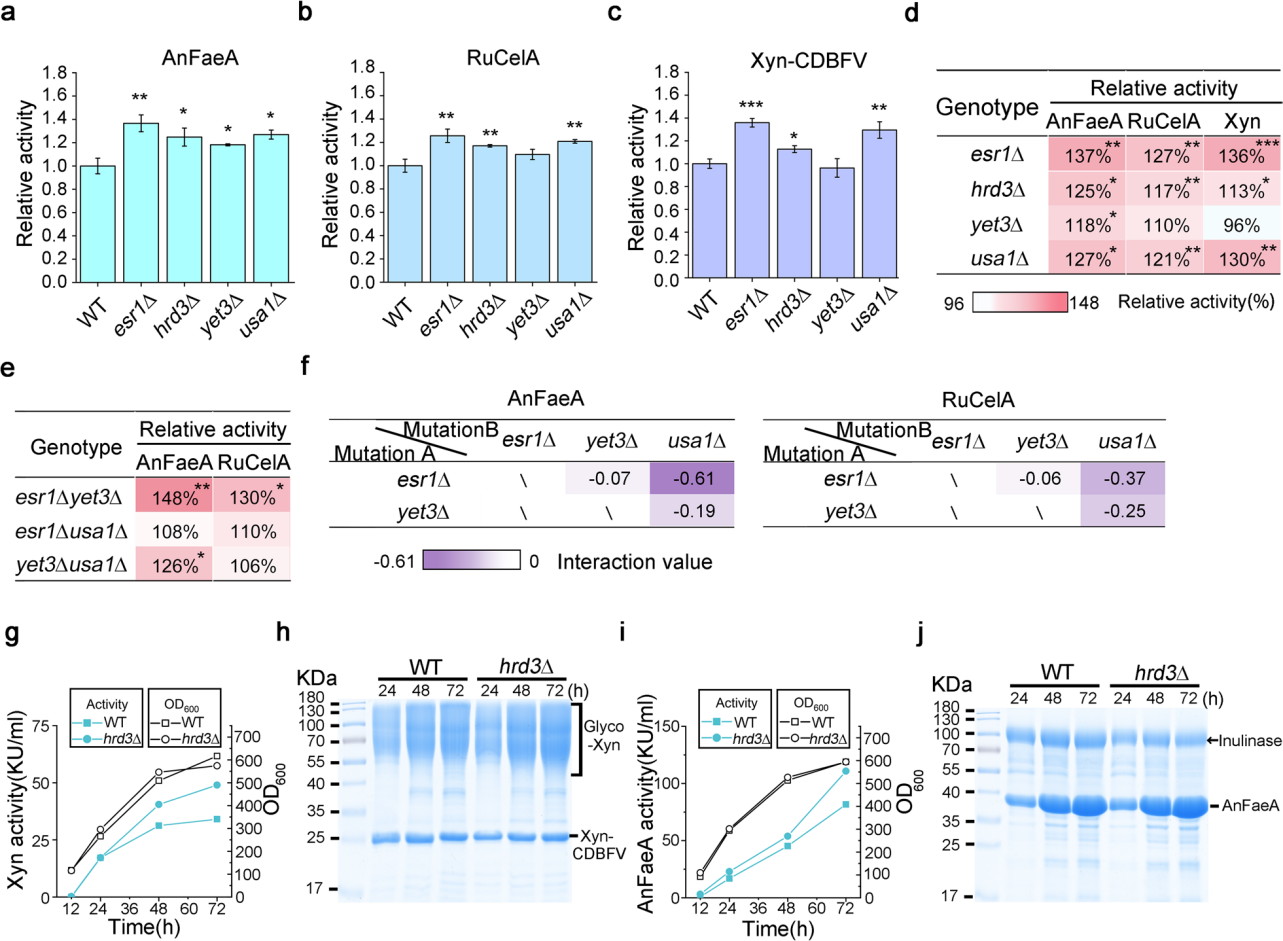
Secretory expressions of lignocellulolytic enzymes in the cells with deletion of ESRTs. Secretory expression of AnFaeA (**a**), RuCelA (**b**) or Xyn-CDBFV (**c**) in the cells with deletion of ESRTs. *ESR1*, *HRD3*, *YET3* or *USA1* was deleted. A multi-copy plasmid overexpressing AnFaeA, RuCelA or XynCDB was transformed into deletion mutants. Extracellular activity in the supernatant was measured. The activity of AnFaeA, RuCelA or Xyn-CDBFV expressed by the wild-type strain was designated as unit 1. Value represented mean ± SD (*n* = 3) (*P < 0.05, **P < 0.01, ***P < 0.001). **d** Summary of effects of deletion of ESRTs. Data were extracted from (a ~ c) (*P < 0.05, **P < 0.01, ***P < 0.001). **e **Extracellular activities of AnFaeA and RuCelA in double-deletion mutants. The activity of AnFaeA or RuCelA expressed by the wild-type strain was designated as unit 1. Value represented mean ± SD (*n* = 3) (*P < 0.05, **P < 0.01). **f** Interaction between deletion of ESRTs. Trait value (*T*) was represented by the relative activity in the double or single deletion mutant compared with that in wild-type cells. The interaction value was calculated as *T*_AB_-*T*_A_-*T*_B_, where *T*_AB_ represented the trait value of a double-deletion mutant, while *T*_A_ and *T*_B_ represented values of single mutants. Interaction values are shown in the chart. Values closing to zero indicated additive interactions and negative values indicated negative interactions. **g**–**j** Expression of Xyn-CDBFV or AnFaeA by wild-type and *hrd3*Δ cells in a 5-L fermentor. Cells were grown in the fermentor for 72 h. Curves of enzymatic activity and OD_600_ were plotted for cells expressing Xyn-CDBFV (**g**) and AnFaeA (**i**). SDS-PAGE analysis of 8 ul supernatant collected at indicated time point is shown in **h**, **j**. The arrow in **j** indicated the position of inulinase, which was a host protein secreted by *K. marxianus* [63]

As shown in Fig. 5c, overexpressing some ESRTs significantly reduced the secretory expressions of AnFaeA or RuCelA. To investigate whether their deletions can promote secretion, *ERS1*, *HRD3*, *YET3* and *USA1*, were deleted in T1, respectively, to obtain LHP1027 ~ LHP1030 (Additional file 5: Table S5). Besides RuCelA and AnFaeA, an endo-1,4-β-endoxylanase Xyn-CDBFV was also overexpressed by a pKD1-based multi-copy plasmid. Xyn-CDBFV undergoes heavy glycosylation in *K. marxianus* [48]. Plasmids expressing RuCelA, AnFaeA or Xyn-CDBFV were transformed into LHP1027 ~ LHP1030 separately. Transformants were grown in flasks and extracellular activities in the supernatant were measured. As shown in Fig. 6a–d, deletion of *USA1*, *HRD3* or *ESR1* significantly improved the expression of RuCelA, AnFaeA and Xyn-CDBFV. To investigate the relationships between *ESR1*, *YET3* and *USA1*, double-deletion mutants, including *usa1*Δ*yet3*Δ, *usa1*Δ*esr1*Δ and *yet3*Δ*esr1*Δ were constructed. Expressions of AnFaeA and RuCelA in a combined mutant were measured and compared with those in a single mutant (Fig. 6e, f). Deletions of *USA1* and *YET3* showed weak negative interaction, suggesting both genes affect the secretion in partially overlapped pathways. This was expected because both *USA1* and *YET3* play roles in ERAD [46, 47]. Deletions of *ESR1* and *YET3* showed additive interaction, suggesting two genes mediated secretory expressions through different pathways. The deletion of *USA1* showed relatively strong negative interactions with the deletion of *ESR1*, suggesting two genes used overlapped pathways to affect secretory expressions. Therefore, *ESR1* might be functionally related to *USA1*. The *esr1*Δ, *hrd3*Δ, *usa1*Δ mutants were genomically stable and were suitable to serve as chassis for the expression of heterologous proteins and construction of consolidated bioprocessing (CBP) strains for bioethanol production.

To evaluate the industrial potential of ESRT mutants for producing lignocellulose-degrading enzymes, episomal plasmids expressing Xyn-CDBFV or AnFaeA were transformed into the *hrd3*Δ mutant and transformants were grown in a 5-L fermentor for 72 h. A synthetic medium was used in the fermentation to reduce cost. During the 72 h fermentation, the OD_600_ curve of *hrd3*Δ cells resembled those of wild-type cells, suggesting the mutation did not affect the growth of cells (Fig. 6g, i). The extracellular amounts of Xyn-CDBFV and AnFaeA in *hrd3*Δ cells were 43% and 28% higher than those of wild-type cells, with the activities of 48,970 U/mL and 14,570 U/mL at 72 h, respectively (Fig. 6g, i). Improved secretions of enzymes in the *hrd3*Δ mutant were confirmed by the SDS-PAGE analysis (Fig. 6 h, j). Based on the specific activities of Xyn-CDBFV (4000 U/mg) and AnFaeA (18,000 U/mg), the protein concentrations of Xyn-CDBFV and AnFaeA in the culture of *hrd3*Δ cells were predicted to be 12.2 g/L and 6.16 g/L, respectively, which were the highest concentrations of endoxylanase and feruloyl esterase produced by *K. marxianus* so far.

## Discussion

In this study, we characterized the transcriptional scope of ER stress in *K. marxianus* for the first time. A total of 892 genes were significantly upregulated upon treatment of DTT or TM. Among upregulated genes, 115 genes were identified as ESRTs and 97 genes as UPR target genes. 58% of ESRTs and 53% of UPR target genes were previously linked with UPR or ER stress, suggesting the majority of the ER response and UPR network was conserved in yeast and fungi. Meanwhile, the rest genes were novel ESRTs and UPR target genes, which provides valuable information for a better understanding of ER response and UPR network in *K. marxianus*. Among the newly identified UPR target genes, four genes encoding subunits of the GID complex, including *VID28*, *VID30*, *FYV10* and *GID8*, were induced rapidly upon DTT treatment. *VID30* and *FYV10* promoters contain UPRE-2 motifs (Additional file 4: Table S4), which are not found in the promoters of their orthologs in *S. cerevisiae*. GID complex is required for the degradation of Fbp1, a key enzyme in gluconeogenesis, and that leads to the metabolic switch from gluconeogenesis to glycolysis [27]. Consistently, glycolytic gene *PGK*, fermentation genes *PDC1* and *ADH1* were induced following the induction of genes encoding GID subunits (Fig. 3b, Additional file 1: Table S1). Gluconeogenesis hydrolyzes four ATPs and two GTPs to direct the process of glucose formation [49]. In contrast, glycolysis and fermentation produce two ATP per glucose with a high rate and low yield, compared to ATP production with a low rate and high yield in respiration [50]. Therefore, one direct outcome of the switch from gluconeogenesis to glycolysis and fermentation is to provide ATP quickly, suggesting ER stress raises an urgent demand for ATP in *K. marxianus*. Overexpressing genes encoding GID subunits and glycolytic enzymes might fasten the flux to glycolysis and help cells to relieve the ER stress more quickly. Consistent with this idea, overexpression of *VID28*, *PDC1* or *PGK* improved expressions of lignocellulolytic enzymes (Fig. 5c). Increased expression of genes involved in glycolysis was in company with decreased expressions of genes involved in cellular respiration, as shown by the downregulation of oxidative phosphorylation genes after 15-min DTT treatment (Fig. 2e, g). The result suggests a preference for the high rate of ATP production during the initial response to ER stress. Interestingly, a shift from respiration to fermentation was observed in a set of *S. cerevisiae* mutants displaying improved secretory expressions of α-amylase [25]. This suggests promoting a flux to glycolysis and fermentation is an effective strategy to improve secretory expressions in yeast.

ERAD is a noteworthy pathway that was related to the secretory expression in this study. In the ERAD-L system, which is best characterized in *S. cerevisiae*, misfolded proteins in the ER lumen are processed, recognized and then transferred to the Hrd1 complex. Hrd1 complex, composed of multispanning ubiquitin ligase Hrd1 and four additional proteins (Hrd3, Der1, Usa1, Yos9), is responsible for the retrotranslocation-coupled ubiquitination of the ERAD substrates. Following retrotranslocation to the cytoplasm, ubiquitinated substrates are transferred to the 26S proteasome via the Cdc48 complex and are degraded afterwards [26]. In this study, genes encoding subunits of the Hrd1 complex, including *HRD1*, *HRD3*, *DER1* and *USA1*, were identified as UPR target genes. Deletion of *USA1* and *HRD3* promoted the secretory expressions of three lignocellulolytic enzymes in the flask using a rich medium. Improved secretory expression in *hrd3*Δ cells was reproduced in a 5-L fermentor. In *S. cerevisiae*, deletion of Hrd1, Hrd3 or Yos9 decreased the secretory expression of a human IgG [51]. In *A. niger*, deletion of Der1 or Hrd3 improved the intracellular amount of GalGus [31]. Therefore, deletions of the Hrd1 complex subunits displayed species-specific effects on the productions of heterologous proteins. Hrd3 is responsible for the initial recognition of glycosylated substrates [38]. Usa1 is required for optimal function and regulation of Hrd1 [52]. Deletion of *HRD3* and *USA1* severely impaired the degradation of ERAD substrates [47]. In *K*. *marxianus*, the degradation of unfolded or misfolded heterologous proteins via the ERAD system might be interfered in *hrd3*Δ and *usa1*Δ cells, and heterologous proteins are cleared from the cells through the alternative secretion route, which improves the secretion of heterologous proteins. Disruption of the Hrd1 complex might serve as a new strategy to improve the secretion expression of heterologous proteins in *K. marxianus*.

In this study, a novel gene, *ESR1*, was identified. There is no ortholog of Esr1 in *S. cerevisiae* and Esr1 in *K. marxianus* shares only 66% identity with its ortholog in the sister species *K. lactis*, suggesting it is a newly evolved protein. ESR1 was an ESRT but not a UPR target gene. Deletion of *ESR1* promoted secretory production of three lignocellulolytic enzymes (Fig. 6d). Deletion of *ESR1* displayed a negative interaction with deletion of *USA1* and an additive interaction with deletion of *YET3* in improving secretory expressions (Fig. 6e). *ESR1* were induced by both DTT and TM (Fig. 3b, Additional file 2: Table S2). Genes displaying similar transcriptional patterns to *ESR1* were enriched in the GO terms of ‘membrane trafficking’ and ‘protein processing in the endoplasmic reticulum’. Moreover, Esr1 was predicted by OrthoDB to be an integral component of the membrane. Taken together, Esr1 might be a protein localized in the ER membrane involved in protein secretion.

Overexpressing *CAJ1* improved the extracellular activities of both AnFaeA and RuCelA. Caj1 belongs to the Hsp40/DnaJ family. DnaJ/Hsp40-like genes were identified as UPR target genes in human [53]. In *S. cerevisiae*, Caj1 colocalizes with the plasma membrane and overexpressing Caj1 stabilizes plasma membrane proteins, including amino acid permeases [45]. Overexpression of Caj1 may stabilize specific plasma membrane proteins required for the export of proteins through the membrane, which leads to improved secretion in *K. marxianus*.

High-level secretory expressions of heterologous cellulases and glycosidases might promote the efficiency of CBP. For example, a semi-industrial *S. cerevisiae* strain displaying improved expressions of heterologous α-amylase and glucoamylase from multi-copy plasmids achieved a 70% increase in the production of ethanol, compared with a laboratory strain expressing the same enzymes [54]. The improved extracellular activity of an integrated inulinase in *S. cerevisiae* increased ethanol production from inulin and Jerusalem artichoke tuber powder in 24 h [55]. In this study, the deletion or overexpression of specific ESRTs of *K. marxianus* improved the secretion of lignocellulolytic enzymes expressed from episomal plasmids or integrated loci. So far, genes encoding heterologous cellulolytic enzymes were integrated into the genome of *K. marxianus* for CBP [56–58]. In the future, it will be intriguing to investigate the effect of modulating ESRTs on the productions of enzymes and the CBP efficiency in these strains.

## Methods

### Strains and plasmids

Strains used in this study are listed in Additional file 5: Table S5. Plasmids are listed in Additional file 6: Table S6. Relevant primers are listed in Additional file 7: Table S7. FIM-1ΔU strain was used as a wild-type strain for RNAseq [48]. T1 strain was used for expressions of lignocellulolytic enzymes [41].

LHZ765 served as a backbone for overexpressing ESRTs. *INU1* promoter and terminator in pUKDN132 were replaced by *TEF* promoter and *TEF* terminator, respectively, to obtain LHZ765. The ORF of ESRTs were inserted between *Sac*II and *Pac*I sites of LHZ765 separately to obtain LHZ767 ~ LHZ880. The ORF of *AnFaeA* was inserted between *Sac*II and *Pac*I sites of LHZ765 to obtain LHZ766. LHZ442 (pZP52) overexpressing RuCelA and LHZ443 (pZP46) overexpressing Xyn-CDBFV were described previously [48].

Deletion of a gene in *K. marxianus* was performed by homologous recombination with the aid of a CRISPR plasmid. Three CRISPR vectors, LHZ296, LHZ301 and LHZ531, were constructed and used as backbones to build CRISPR plasmids (Additional files 8, 9, 10). pUKD-N122-AUC contains two *Sap*I sites for the insertion of a target sequence in front of gRNA [41]. The ARS7 fragment in pUKD-N122-AUC was replaced by a pKD1 fragment [59], to obtain LHZ296. A second gRNA expression cassette containing two *Aar*I sites for the insertion of the target sequence was cloned upstream of *KmURA3* in LHZ296 to construct LHZ301. The pKD1 fragment in LHZ296 was replaced by ARS1/CEN [60], to construct LHZ531. Primers containing 20 bp target sequence were annealed in pairs and inserted into *Sap*I or *Aar*I sites of LHZ296, LHZ301 and LHZ531. Details of resultant CRISPR plasmids are listed in Additional file 6: Table S6.

To delete *IRE1* in FIM-1ΔU, *IRE1* with the flanking sequence was amplified and ligated with pMD18-T. The ORF of *IRE1* in the resulting plasmid was removed by mutagenesis PCR to obtain LHZ748. Donor sequence was amplified from LHZ748 and co-transformed with CRISPR plasmid LHZ747 into FIM-1ΔU to obtain LHP1019. Similarly, *HAC1* was deleted in FIM-1ΔU to obtain LHP1020. *ESR1, HRD3, YET3* and *USA1* were deleted in T1, respectively, to obtain LHP1027 ~ LHZ1030. *ESR1* was deleted in LHP1029 and LHP1030 to obtain LHP1031 and LHP1032, respectively. *YET3* was deleted in LHP1030 to obtain LHP1033.

To replace *INU1* with a gene of interest, *INU1* with the flanking sequence was amplified and ligated with pMD18-T. The ORF of *INU1* in the resulting plasmid was removed by mutagenesis PCR to obtain LHZ424. To replace *INU1* with *AnFaeA*, the ORF of *AnFaeA* was amplified and inserted between *Spe*I and *Not*I sites of LHZ424. The donor sequence was amplified from the resultant plasmid and co-transformed with LHZ759 into T1 to obtain LHP643. Similarly, *INU1* in T1 was replaced by *RuCelA* to obtain LHP1021.

For enzymatic assays, plasmids overexpressing different ESRT (LHZ767 ~ LHZ880) was transformed into LHP643 and LHP1021. The plasmid overexpressing Xyn-CDBFV (LHZ443) was transformed into LHP1027 ~ LHP1031. The plasmid overexpressing RuCelA (LHZ442) or AnFaeA (LHZ766) was transformed into LHP1027 ~ LHP1033. Transformants were selected on Sc-Ura medium.

### Media

*K. marxianus* cells were cultivated at 30°. C. YPD medium (2% peptone, 1% yeast extract, 2% agar for plates), synthetic complete minus uracil medium (SC-Ura) and synthetic dextrose minimal medium (SD) were prepared as described before [61]. YG liquid medium (4% glucose, 2% yeast extract) was prepared for expressions of AnFaeA, RuCelA and Xyn-CDBFV in flasks.

### Spot assay

Fresh FIM-1ΔU, LHP1019 or LHP1020 cells were grown in YPD liquid medium overnight. Cells were collected and adjusted to an OD_600_ of 1.0. Then, fivefold serial dilutions were performed and 5 µL dilutions were spotted onto YPD containing 10 ~ 30 mM DTT (D8220, Solarbio, China) or 0.1 ~ 0.5 µg/mL TM (T8480, Solarbio). The plates were incubated at 30 °C for 2 days.

### RNA extraction and qPCR

Fresh FIM-1ΔU and LHP1020 cells were grown in YPD liquid medium overnight. Cells were diluted into a fresh YPD liquid medium to start at an OD_600_ of 0.2 and were grown for 3 ~ 5 h until the exponential phase (OD_600_ of 0.6–0.8). The cultures were supplemented with DTT (a final concentration of 10 mM) or TM (a final concentration of 0.5 µg/mL). FIM-1ΔU cells were harvested at 0, 15, 30 and 60 min after treatment. LHP1020 cells were harvested at 15 and 60 min after treatment. Cells were frozen at − 80 °C. Three biological repeats were collected at each time point. RNA was extracted from frozen cells using a Quick-RNA Fungal/Bacterial Miniprep kit (R2010, Zymo Research, USA) and were reverse transcribed using a PrimeScript RT Reagent Kit (RR037A, Takara, China). The qPCR was performed using TB Green Premix Ex Taq (RR820A, Takara). Primers used in qPCR are listed in Additional file 7: Table S7.

### RNA sequencing

RNA was extracted as described above. Samples were reversed transcribed and sequenced by Illumina HiSeq X Ten system at Biomedical Big Data Center, Shanghai Institutes for Biological Sciences, or by Illumina NovaSeq 6000 system at Mingma Biotechnology. A total of 5.6 million reads and 40 million reads were obtained on average for samples of FIM-1ΔU and LHP1020, respectively. Raw sequencing data were uploaded to NCBI. Reads were aligned, assembled and analyzed as previously described [62], by using the genome of *K. marxianus* DMKU 3–1042 as a reference (GenBank assembly accession GCA_001417885.1). A q-value threshold of 0.05 was set in terms of altered expression. At an indicated time point, the relative transcript abundance of a gene was calculated by dividing the Fragments Per Kilobase of exon model per Million mapped fragments (FPKM) value in the cells treated with DTT or TM by the FPKM value in the cell without treatment. The relative transcript abundances of genes are listed in Additional file 1: Table S1.

### Identification of ESRTs and UPR target genes

Orthologs of classic UPR target genes that displayed specific transcriptional patterns during ER stress were selected as reference genes. *DER1*, *HRD1* and *UBC7* were references for the group of genes induced rapidly upon DTT treatment (Group A). *KAR2*, *LHS1*, *ERO1*, *MPD1* and *PDI1* were references for the group of genes gradually induced upon DTT treatment (Group B). *KAR2*, *ERO1* and *PDI1* were references for the group of genes gradually induced upon TM treatment (Group C). *SEC12*, *SEC24*, *SFB3* and *HRD3* were references for the group of genes induced late upon TM treatment (Group D). In each group, the difference of the relative transcript abundance (log_2_) of a reference gene between adjacent time points (15 min-0 min, 30 min-15 min or 60 min-30 min) was calculated. Mean (${\mu }_{i}$) and variance (${\nu }_{i}$) of the difference between reference genes were calculated for each group. Chi-square tests were performed to identify potential ESRT genes based on the formula:

$${\chi }^{2}=\sum_{i=1}^{3}\frac{({x}_{i}-{\mu }_{i}{)}^{2}}{{\nu }_{i}},$$

${x}_{i}$ represented the difference of the relative transcript abundance (log_2_) of a gene of interest between adjacent time points. ${\chi }^{2}$ statistic was calculated by using ${\mu }_{i}$ and ${\nu }_{i}$ of each group with the assumption that the distribution of observation ${x}_{i}$ had a normal distribution. Under the null hypothesis, the ${\chi }^{2}$ statistic follows a central Chi-square distribution with a degree of freedom 3. *P*-value was obtained according to ${\chi }^{2}$. Because DTT induced an acuter ER stress than TM, a high threshold of *P*-value was applied to identify ESRTs induced by DTT in group A and B, while a low threshold of *P*-value was applied to identify UPR targets genes induced by TM in group C and D. A gene with a *P*-value > 0.1 and a fold change > 4 at 15 min during DTT treatment was identified as an ESRT of group A. A gene with a *P*-value > 0.1 was identified as an ESRT of group B. A gene with a *P*-value > 0.05 was identified as an ESRT of group C or D. Two-tailed T-tests were performed to determine if there is a significant difference between the means of relative transcript abundance of a given ESRT in FMI1ΔU cells and *hac1*Δ cells. A timepoint displaying the highest relative transcript abundance in each group was chosen for the T-test, including 15-min DTT treatment in group A, 60-min DTT treatment in group B, 60-min TM treatment in group C and D. An ESRT with a *P*-value < 0.05 was designated as a UPR target gene. A full list of ESRTs and UPR target genes is shown in Additional file 4: Table S4.

### GO analysis

Gene ontology (GO) analyses were performed in each group of genes, respectively, via R (version 4.0.5) with R packages and RSQLite (version 2.2.5), clusterProfiler (version 3.18.1), Rgraphviz (version 2.34.0), pathview (version 1.30.1) and org.Sc.sgd.db (version 3.12.0).

### Enzyme activity assay

Transformants that contained the plasmid overexpressing ESRT, Xyn-CDBFV, RuCelA or AnFaeA were grown on the SC-Ura medium for one day. Then, fresh cells were grown in a YG medium for 72 h. The activities of RuCelA, AnFaeA and Xyn-CDBFV in the supernatant were measured as described previously [48].

### Fed-batch fermentation

Plasmid overexpressing RuCelA (LHZ442) or Xyn-CDBFV (LHZ443) was transformed into LHP1028. Seed culture was prepared by growing fresh transformant in SD liquid medium for 16 h. Fed-batch fermentations were carried out in a 5-L bioreactor (BXBIO, Shanghai, China) as described previously [48]. The supernatant was collected every 24 h. Samples were subjected to the SDS-PAGE analysis and enzymatic assay.

## Supplementary Information

**Additional file 1: Table S1.** Changes of gene expressions during treatment of DTT or TM.

**Additional file 2: Table S2.** Differentially expressed genes upon treatment of DTT or TM.

**Additional file 3: Table S3.** GO analysis of differentially expressed genes upon treatment of DTT or TM.

**Additional file 4: Table S4.** ESRTs and UPR target genes.

**Additional file 5: Table S5.** Strains used in this study.

**Additional file 6: Table S6.** Plasmids used in this study.

**Additional file 7: Table S7.** Primers used in this study.

**Additional file 8.** Sequence of LHZ296 in genbank format.

**Additional file 9.** Sequence of LHZ301 in genbank format.

**Additional file 10.** Sequence of LHZ531 in genbank format.

**Additional file 11: Figure S1.** Box plot of distributions of fold changes of transcript abundance under different treatment conditions.

## Data Availability

The dataset supporting the conclusions of this article is available in the NCBI (https://www.ncbi.nlm.nih.gov/bioproject/PRJNA729880; https://www.ncbi.nlm.nih.gov/bioproject/PRJNA777750).

## Abbreviations

Bp: Base pair
CBP: Consolidated bioprocessing
DTT: Dithiothreitol
ER: Endoplasmic reticulum
ERAD: ER-associated protein degradation
ERS1: ER stress responsible gene 1
ESRTs: ER stress response target genes
TM: Tunicamycin
ORF: Open reading frame
UPR: Unfolded protein response
UPRE: Unfolded protein response element
