# Supplementary material for: Characterization and modulation of endoplasmic reticulum stress response target genes in Kluyveromyces marxianus to improve secretory expressions of heterologous proteins
Source: Biotechnol Biofuels. 2021 Dec 14;14:236. doi: 10.1186/s13068-021-02086-7 (PMC8670139; doi:10.1186/s13068-021-02086-7)
Supplement: Supplementary file 11 — Additional file 11: Figure S1. Box plot of distributions of fold changes of transcript abundance under different treatment conditions. [file 13068_2021_2086_MOESM11_ESM.docx]

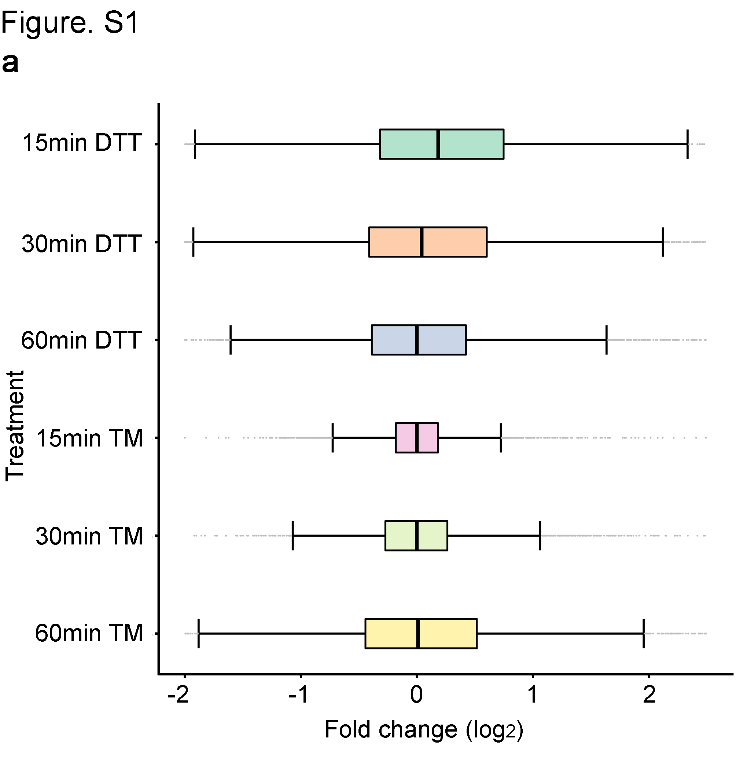


Figure S1. Box plot of distributions of fold changes of transcript abundance under different treatment conditions.

Box plot represented medians and interquartile ranges (IQR) of the Log2 fold changes of transcript abundance in different conditions of treatments. The fold change of a gene was the ratio of the FPKM value in the treated cells to that in cells without treatment. Whiskers were drawn up to the largest and lowest observed points within the distance of 1.5 times IQR above the upper quartile and below the lower quartiles, respectively. All other values are plotted as outliers.
